# Phytochemical Composition and Antioxidant and Anti-Inflammatory Activities of *Humboldtia sanjappae* Sasidh. & Sujanapal, an Endemic Medicinal Plant to the Western Ghats

**DOI:** 10.3390/molecules28196875

**Published:** 2023-09-29

**Authors:** Jameema Sidhic, Satheesh George, Ahmed Alfarhan, Rajakrishnan Rajagopal, Opeyemi Joshua Olatunji, Arunaksharan Narayanankutty

**Affiliations:** 1 Phytochemistry and Pharmacology Division, PG & Research Department of Botany, St. Joseph’s College (Autonomous), Calicut 673008, India; 2 Department of Botany and Microbiology, College of Science, King Saud University, P.O. Box 2455, Riyadh 11451, Saudi Arabia; alfarhan@ksu.edu.sa (A.A.); rrajagopal@ksu.edu.sa (R.R.); 3 African Genome Center, Mohammed VI Polytechnic University, Ben Guerir 43150, Morocco; 4 Division of Cell and Molecular Biology, PG & Research Department of Zoology, St. Joseph’s College (Autonomous), Calicut 673008, India

**Keywords:** *Humboldtia sanjappae*, LC-MS analysis, radical scavenging, anti-inflammatory activity, cytokine release

## Abstract

Ethnomedicinal plants are important sources of drug candidates, and many of these plants, especially in the Western Ghats, are underexplored. *Humboldtia*, a genus within the Fabaceae family, thrives in the biodiversity of the Western Ghats, Kerala, India, and holds significant ethnobotanical importance. However, many *Humboldtia* species remain understudied in terms of their biological efficacy, while some lack scientific validation for their traditional uses. However, *Humboldtia sanjappae*, an underexplored plant, was investigated for the phytochemical composition of the plant, and its antioxidant, enzyme-inhibitory, anti-inflammatory, and antibacterial activities were assessed. The LC-MS analysis indicated the presence of several bioactive substances, such as Naringenin, Luteolin, and Pomiferin. The results revealed that the ethanol extract of *H. sanjappae* exhibited significant in vitro DPPH scavenging activity (6.53 ± 1.49 µg/mL). Additionally, it demonstrated noteworthy FRAP (Ferric Reducing Antioxidant Power) activity (8.46 ± 1.38 µg/mL). Moreover, the ethanol extract of *H. sanjappae* exhibited notable efficacy in inhibiting the activities of α-amylase (47.60 ± 0.19µg/mL) and β-glucosidase (32.09 ± 0.54 µg/mL). The pre-treatment with the extract decreased the LPS-stimulated release of cytokines in the Raw 264.7 macrophages, demonstrating the anti-inflammatory potential. Further, the antibacterial properties were also evident in both Gram-positive and Gram-negative bacteria. The observed high zone of inhibition in the disc diffusion assay and MIC values were also promising. *H. sanjappae* displays significant anti-inflammatory, antioxidant, antidiabetic, and antibacterial properties, likely attributable to its rich composition of various biological compounds such as Naringenin, Luteolin, Epicatechin, Maritemin, and Pomiferin. Serving as a promising reservoir of these beneficial molecules, the potential of *H. sanjappae* as a valuable source for bioactive ingredients within the realms of nutraceutical and pharmaceutical industries is underscored, showcasing its potential for diverse applications.

## 1. Introduction

Humans have always battled with various infections. In addition to these, recent decades have witnessed a significant increase in the occurrence of various non-communicable diseases. These diseases have been associated with increased mortality globally. The changes in lifestyle comprising dietary changes and reduced physical activity have resulted in a sudden increase in the number of patients. The role of oxidative stress and inflammation is impeccable in the onset of these diseases. Oxidative stress, the imbalance between the generation of reactive molecules and its scavenging, plays significant roles in non-communicable diseases [1]. Together with this, numerous studies have established that inflammation has a role in the progression of different diseases. The link between inflammatory response and the onset of different cancers such as ovarian cancer and pancreatic cancer has been studied well [2,3]. Recent studies suggest that neuroinflammation in Alzheimer’s will escalate the disease progression Recently, studies on *Gynostemma pentaphyllum* (Thunb.) Makino demonstrated a protective effect against the inflammation [4,5]. New therapies indicate that the anti-inflammatory treatments associated with cardiovascular disease are a promising strategy to bring down the succession of the disease [6]. Inflammatory processes in the host defense system should be highly regulated, and the loss of control is problematic. So, the inflammatory molecules have become the primary target for the prevention of various diseases, in which the main signaling pathways such as the nuclear factor-κB (NF-κB) signaling pathway, Janus kinase/signal transducer and activator of transcription (JAK/STAT) signaling pathway, and mitogen-activated protein kinase (MAPK) signaling pathway might be brought under control to prevent the diseases [7,8,9]; the JAK/STAT is focused on more because this pathway is associated with the pathogenesis of different inflammatory diseases such as Rheumatoid Arthritis (RA) and Inflammatory Bowel Disease (IBD) [10,11]. Even though inflammation is an evolutionarily conserved mechanism for the organism’s survival [12], it is essential to control the prolonged release of anti-inflammatory mediators to prevent the development of various diseases [13]. Different natural products have recently been investigated and have given satisfactory results in this respect [14,15]. Excess inflammation in the body will lead to the development of Reactive Oxygen Species (ROS); if the concentration of the same exceeds a limit, the body will not be able to neutralize the same [16]. Due to this reason, pharmaceutical and food industries have considerably used natural products with antioxidant capabilities, and herbal products that promote health have also become highly popular in recent years [17,18].

It has been discovered that several plant medicines have a variety of pharmacological properties, including antioxidant, anti-inflammatory, anticancer, neuroprotective, and hypolipidemic properties [8,19,20]. These activities are attributed to the non-nutritive chemicals present in the plants [10]. Findings from studies conducted in mice suggest that the leaves of *Hemigraphis alternata* exhibit anti-inflammatory, anti-nociceptive, and anti-diarrheal activities [21]. These kinds of phytochemicals are reported in several plant families such as Lamiaceae, Zingiberaceae, Malvaceae, Acanthaceae, and Apocyanacea [22,23]. Fabaceae is one such family with an abundance of different phytochemicals which are good in curing various diseases [24].

The genus *Humboldtia* belongs to the family Fabaceae. The plants of the particular genus are well known for their traditional uses and pharmacological properties [25]. Research has been conducted on certain species of *Humboldtia*, revealing their rich phytochemical composition. These plants have been found to contain valuable phytochemicals, including phenols, flavonoids, saponins, tannins, terpenoids, cardiac glycosides, apigenin, steroids, phlobatannins, and more [26,27,28]. In the realm of traditional medicine, the bark of *Humboldtia* species held curative significance, being employed to address conditions such as biliousness, leprosy, ulcers, and epilepsy and acting as an anticonvulsant [29]. The remediation of biliousness, impurities in the blood, ulcers, and epilepsy all involved the preparation of a decoction from the bark powder [30].

*Humboldtia brunonis* Wall, *Humboldtia unijuga* Bedd., and *Humboldtia vahliana* Wight are well known for their pharmacological efficiency and their antioxidant, anti-inflammatory, anticancer, antimicrobial, and anti-depressant effects [28,29,31,32,33]. *H. brunonis* fulfilled roles as a styptic, demulcent, anthelmintic, ulcer remedy, stomachic, astringent, and treatment for menstrual and urinary issues [34]. Furthermore, the local populations residing in Karnataka’s Shiradi and Bisle Ghats harnessed the leaves and bark of *H. brunonis* for arthritis and diabetes treatments, a practice detailed by Prasad and Kumar [26]. It was documented that the *H. brunonis* bark and leaves were utilized for addressing wounds, menstruation disorders, and overgrowth issues [35]. *Humboldtia unijuga*, referred to as ‘palakan’ by the Kani tribes in Agasthyamala, was employed to treat ailments such as headaches, chickenpox, and snake bites [36]. It has been discovered that the plant possesses Erythrodiol-3-acetate and 2,4-di-tert-butylphenol, which have been demonstrated to exhibit anti-inflammatory and anticancer properties [25].

The plant species *Humboldtia sanjappae*, belonging to the Fabaceae family, and its related species exhibit a diverse range of pharmacological properties [28,29,31,32,33]. However, the phytochemical constituents and pharmacological effects of this plant remain largely unexplored. Currently, there are no existing reports on the antioxidant and anti-inflammatory activities of this plant, and data regarding its phytochemical composition are also lacking. Consequently, this study represents a pioneering effort to investigate the phytochemical profile of the plant and evaluate its potential antioxidant, enzyme-inhibitory, anti-inflammatory, and antibacterial properties. To identify the bioactive phytochemicals, LC-MS analysis is employed as a key analytical technique.

## 2. Results and Discussion

### 2.1. Quantitative and Qualitative Estimation of Phytochemicals in H. sanjappae

**Figure 1:**
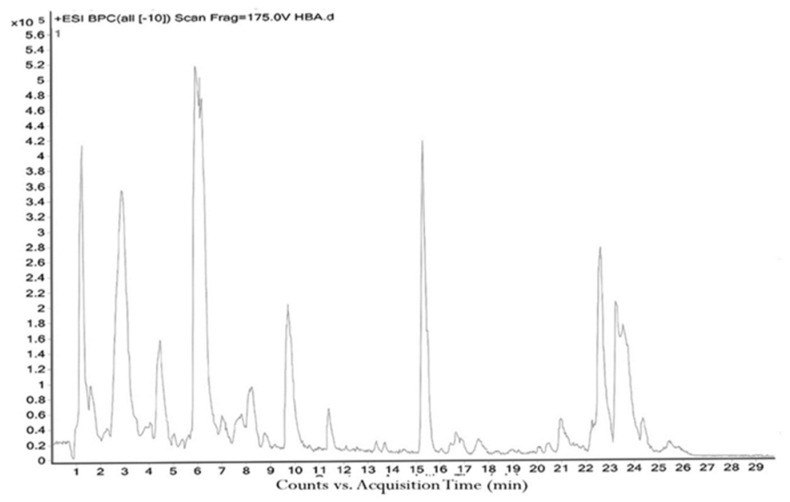
The LC−MS total ion chromatogram of *H. sanjappae* ethanol extract.

The *H. sanjappae* extract was analyzed using LC-MS, which indicated the presence of flavonoids, including Naringenin, Luteolin, and Pomiferin, as well as phenols such as Epicatechin and Maritemin (Figure 1, Table 1). Flavonoids and phenolic substances not only have antioxidant properties, but they also work well as anti-inflammatory agents [37]. Various studies provide support for the immune-modulating effects of polyphenols and flavonoids. Seed polyphenols extracted from *Nigella sativa* L. were evaluated for their analgesic and anti-inflammatory properties. The study findings demonstrated that these polyphenols effectively reduced paw edema induced by carrageenan [38]. Concentrated extract derived from *Dendrobium loddigesii* Rolfe, rich in polyphenols, was administered to treat diabetic mice. The outcomes indicated that this extract exhibited the capability to lower blood glucose levels, reduce body weight, decrease levels of low-density lipoprotein cholesterol, and elevate insulin levels within the mice [39]. Flavonoids are part of the category of polyphenolic natural compounds, encompassing over 4000 identified variations. The advantageous biological effects of flavonoids are unquestionably intertwined with their structural composition and properties, rendering them prime contenders for pharmaceutical development. Numerous inflammatory molecules such as TNF-α, IL-1, IL-6, IL-17, and IFN-γ, released via the activation of various signaling pathways, primarily the NF-κB pathway, have been demonstrated to be inhibited upon administration of flavonoid [40]. Scientists detected that supplementation of Epicatechin potentially contributed to reducing inflammation and enhancing insulin sensitivity in visceral adipose tissue of high-fat fed mice [41]. The presence of phytochemicals such as Epicatechin in the plant may be responsible for its observed antidiabetic activity. However, additional experiments and studies are necessary to validate this hypothesis and confirm the specific compounds and mechanisms involved in the plant’s potential benefits for diabetes management. Certainly, the antioxidant properties of the extract can play a crucial role in managing oxidative stress in individuals with diabetes. As diabetes can cause substantial cellular damage, including in the brain, combating oxidative stress with antioxidant compounds becomes important [42]. By reducing oxidative stress, the extract’s antioxidant properties may help protect cells, mitigate damage, and contribute to improved overall health in diabetic patients.

Several investigators have noted natural substances’ anti-inflammatory properties, including multiple preclinical experiments [43,44,45,46]. As a result, we infer that the antioxidant and anti-inflammatory properties exhibited by the ethanol extract of *H. sanjappae* must be due to the presence of various phytochemical components such as polyphenols, flavonoids, isocoumarins, etc., and also that these many different phytochemicals in plants offer a valuable source of antimicrobial compounds with immense therapeutic potential [47]. Antibiotic-resistant bacteria have emerged as a significant global health concern [48,49]. Plants have long been renowned for their antibacterial powers as nature’s medicine [50]. Polyphenols and essential oils, among other bioactive substances, have powerful antibacterial properties [30,51,52,53]. Adopting plant phenomena might open the way for innovative and long-lasting antibacterial treatments because traditional antibiotics are becoming less effective, leading to a pressing need for the development of new and effective antimicrobial agents. In this context, the rich diversity of plant species provides a vast array of bioactive compounds that can be explored for their antimicrobial properties. Polyphenols and flavonoids possess well-documented antimicrobial properties [54,55], exhibiting inhibitory effects against a broad spectrum of bacteria [55,56]. 

Our research has supplied substantiating proof of the elevated levels of these compounds in the ethanol extract of *H. sanjappae*. Specifically, the total phenolic content measured at 378.77 ± 6.62 µg equivalent per milligram and the total flavonoid content recorded at 204.76 ± 6.10 µg equivalent per milligram both emphasize the concentration within the extract (Table 2). Given its rich content of polyphenols and flavonoids, the extract from *H. sanjappae* shows promise as a potential source for the development of novel antibiotics. Considering its antimicrobial potential, the polyphenol- and flavonoid-rich extract of *H. sanjappae* warrants further exploration in the search for new antibiotic compounds. 

### 2.2. In Vitro Antioxidant Activities of H. sanjappae Extract

Species of the Fabaceae family consist of phytochemicals responsible for the plant’s antioxidant potential [57]. The different genera of the family are established as having antioxidant potential [58]. In vivo studies of *Tamarindus*, a related genus of *Humbodtia*, showed that it exhibits potent antioxidant activity [59], and the antioxidant potential of species within the genus *Humboldtia* has been previously explored, and their effectiveness has been reported [27,31]. The IC_50_ value of *H. sanjappae* bark extract in the anti-DPPH radical scavenging assay was shown to be 6.53 ± 1.49 µg/mL. Likewise, Table 3 shows other antioxidant activity in Ferric Reducing Antioxidant Power, represented by its value of 8.46 ± 1.38 µg/mL. The antioxidant properties of the plant must be assigned to the different phytochemicals present in the extract; those bioactive compounds identified from LC-MS analysis are listed in the Table 1. For example, previous studies demonstrated that anticancer properties of Epicatechin are linked to its antioxidative potential [60]. Another component present in the extract is Luteolin, which is found in glycosylated forms in a variety of vegetables and fruits and is classified within the flavone subclass of flavonoids. Its documented effects include in vivo anti-inflammatory [61], antioxidative [62,63,64], antidiabetic [61], antimicrobial [65], and anticancer [66,67] activities. The antioxidant properties of Naringenin [68,69], Morindone [70,71], Capsanthin 5,6-epoxide [72,73], and Ganoderic acid F [74] have been previously established. Therefore, these compounds could potentially account for the robust antioxidant activity observed in the extract. Oxidative stress plays a critical role as an independent factor in the development of numerous chronic diseases, including cancer, diabetes, and cardiovascular diseases [75,76,77,78]. Therefore, the antioxidant properties found in the plant extract can be beneficial in the management of diseases that are linked to oxidative stress. 

### 2.3. Enzyme Inhibitory Properties of H. sanjappae Ethanol Extract

The extract was examined for its enzyme-inhibitory properties against key enzymes associated with type 2 diabetes mellitus, namely α-amylase and α-glucosidase. The IC_50_ value for the inhibition of α-amylase and α-glucosidase by the extract was determined to be 47.60 ± 0.19 µg/mL and for the inhibition of β-glucosidase, 32.09 ± 0.54 µg/mL (Table 3). The standard antidiabetic drug acarbox exibited an IC_50_ value of 122.18 ± 3.08 and 103.45 ± 2.68 µg/mL against α-amylase and α-glucosidase enzymes, respectively. Hence, the plant extract contains stronger antidiabetic compounds compared to the acarbose. The α-amylase and α-glucosidase are enzymes that play crucial roles in carbohydrate metabolism and are frequently targeted by antidiabetic medications [79]. Indeed, the inhibition of α-amylase and α-glucosidase by the *H. sanjappae* extract may contribute to its potent antidiabetic activity. By inhibiting these enzymes involved in carbohydrate metabolism, the extract can potentially help regulate blood glucose levels and manage diabetes effectively. The enzyme-inhibitory properties are well corroborated by the major bioactive substances observed in the plant using LC-MS analysis. Epicatechin, Luteolin, and Naringenin were reported to inhibit the α-amylase and α-glucosidase in in vitro and animal model studies [80,81]. In addition, the reports clearly indicated the antidiabetic properties of these bioactive compounds in independent studies.

### 2.4. Anti-Inflammatory Activity of H. sanjappae

The anti-inflammatory activity of the extract was evaluated using Raw 264.7 macrophages as the model. Raw 264.7 cells stimulated with lipopolysacchride (LPS) are a widely utilized cellular model of inflammation [82]. The lipopolysaccharide is the cell wall component of most of the Gram-negative bacteria; the LPS stimulates the macrophage in a toll-like-receptor-dependent manner [83]. In the present study, the normal macrophage was estimated for the level of IL-1β, and it was estimated to be 64.6 ± 1.9 pg/mg protein. However, there was observed a significant elevation in the IL-1β levels upon stimulation with the lipopolysaccharide to 573.4 ± 4.5 pg/mg protein. The increased level of IL-1β is an indicator of inflammation in the cellular conditions [84,85,86]. However, the pre-treatment of macrophages with the different doses of HSE resulted in a significant reduction in IL-1β levels (Table 4). The pre-treatment of Raw 264.7 cells with 5 µg/mL of HSE resulted in cellular IL-1β levels of 403.7 ± 6.2 pg/mg protein (*p* < 0.01). Similarly, the pre-treatment with 10 µg/mL (298.5 ± 8.4 pg/mg protein) and 20 µg/mL of HSE (156.2 ± 3.4 pg/mg protein) resulted in lower IL-1β levels (*p* < 0.001). The reduction in IL-1β levels is indicative of the anti-inflammatory potential of the HSE at the respective treatment doses.

Together with IL-1β, IL-6 was also found to significantly influence the inflammation in macrophages [87,88]. IL-6 has a major role in the innate immune defense systems [89]; however, the same molecule is associated with the progression of various diseases [90,91]. The level of IL-6 in the untreated macrophages without LPS stimulation was estimated to be 133.4 ± 5.8 pg/mg protein; however, the exposure of LPS elevated the cellular IL-6 levels to 628.5 ± 8.2 pg/mg protein. On the contrary, the level was brought down by the treatment with 5 and 10 µg/mL of *H. sanjappae* extract, which reduced the cellular IL-6 levels to 507.1 ± 8.1 (*p* < 0.05) and 388.4 ± 2.8 pg/mg protein (*p* < 0.001). In the highest concentration of *H. sanjappae* extract treatment, the IL-6 was estimated to be 291.3 ± 6.6 pg/mg protein (*p* < 0.001).

The TNF-α levels are crucial for the survival and proliferation of various cancer cells [92]. The cytokine is also important in the progression events of cancers including metastasis and stemness [93]. The level of TNF-α in the untreated and unstimulated Raw 264.7 macrophages was estimated to be 115.2 ± 3.1 pg/mg protein. However, the level was elevated to 856.0 ± 11.2 pg/mg protein upon stimulation by the LPS. This clearly indicated the induction of acute inflammation in the experimental condition. In 5 µg/mL *H. sanjappae* treated macrophages, the level of TNF-α was reduced to 715.2 ± 8.8 pg/mg protein. A similar decrease in the TNF-α level was also noted in the 10 and 20 µg/mL *H. sanjappae* treatment, which brought down the TNF-α level to 602.8 ± 5.2 and 493.7 ± 6.4 pg/mg protein.

The nitric oxide level is also an important inflammatory indicator in cells; the inducible nitric oxide synthase is an enzyme responsible for the overwhelming load of nitric oxide in the body [94]. Despite its physiological and immunological importance, nitric oxide is often associated with chronic inflammation and is thereby involved in many of the degenerative diseases [95]. The level of nitric oxide in the untreated macrophage cells was estimated to be 10.7 ± 0.64 µM/mg protein. The level was increased to 75.2 ± 2.1 µM/mg protein in the macrophages exposed to LPS. Interestingly, the level was brought down by the pre-treatment with the 5 µg/mL of HSE (58.8 ± 3.4 µM/mg protein). In the 10 µg/mL of *H. sanjappae* extract treatment, the NO level was estimated to be 42.3 ± 1.9 µM/mg protein, and, in the 20 µg/mL HSE treatment, it was further reduced to 30.7 ± 2.5 µM/mg protein. Hence, it is clearly indicated that the pre-treatment with different doses of HSE dose-dependently reduced the inflammatory insults in cultured macrophages.

Pathogen-associated molecular pattern molecules (PAMPs) or damage-associated molecular pattern molecules (DAMPs) are the two types of molecules that trigger the production and release of IL-1β. LPS is the main outer surface membrane component and is a highly potent PAMP which stimulates innate or natural immunity in various eukaryotic cells [96]. LPS-induced inflammatory responses are linked to the production of ROS in cells [97]. *H. sanjappae* extract was found to possess anti-inflammatory potential in a dose-dependent manner (Table 4). In vitro analysis revealed that it inhibits nitric oxide (NO) radicals. The inflammatory insults caused by LPS are prevented in cells pre-treated with *H. sanjappae* extract, and cytokine level is also reduced as a result. Interleukin-1β (IL- 1β) is a potent proinflammatory cytokine vital in the host cell defense reaction to infection [98]. After LPS stimulation, macrophages were shown to have a considerably higher level of IL-1β; however, pre-treatment with *H. sanjappae* extract at various dosages dramatically reduced the IL-1β levels in the macrophages. Like IL-β, interleukin-6 (IL-6) and tumor necrosis factor-α (TNF-α) are significant mediators of innate immunity [99]. LPS also elevated the level of these cytokines. Application of the *H. sanjappae* extract reduced the elevated levels of TNF-α and IL-6.

The bioactive flavonoids present in the *H. sanjappae* extract such as Epicatechin, Luteolin, and Naringenin might play an important role in the anti-inflammatory potential of the plant. Independent studies have reported the anti-inflammatory potential of these bioactive flavonoid molecules in cultured macrophages and animals [37].

**Figure 2:**
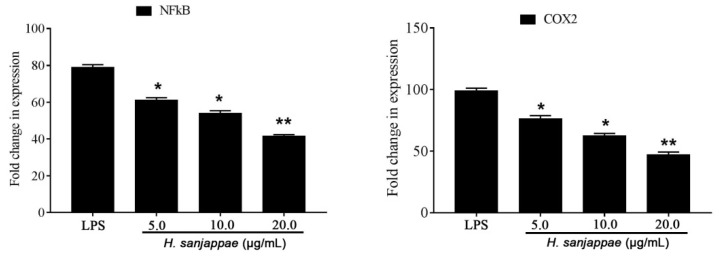
The expression of NF-KB and COX2 genes in the Raw 264.7 macrophages treated with LPS and different doses of *H. sanjappae* leaf methanol extract. * indicates significant variation with respect to LPS control (*p* < 0.05); ** indicates higher significant variation with respect to that of LPS control (*p* < 0.01).

To further explain the mechanism of anti-inflammatory activity, the expression of genes NF-KB and COX2 was assessed. Compared to the untreated LPS control, the *H. sanjappae* extract treatment significantly brought down the expression of NF-KB and COX2. The expression of NF-KB is a crucial event in inflammation, and it is associated with various diseases including cancers. Likewise, the COX2 is a well-known inflammatory enzyme associated with the production of prostaglandins. The expression of COX2 is also evident in different forms of cancers (Figure 2).

### 2.5. Antibacterial Activity of H. sanjappae

The antibacterial activity of *H. sanjappae* is being documented for the first time, while antimicrobial properties of *H. brunonis* have previously been reported [100]. Previous studies have investigated the antimicrobial potential of different extracts from *H. brunonis*, where the methanolic extract of the leaves [101] and aqueous extract of stem and leaf [33] exhibited significant antibacterial activity. It is worth noting that many species within the genus have not been extensively studied in this regard. Therefore, there remains a considerable knowledge gap regarding the antimicrobial potential of the majority of species within the genus. The present study observed a significant antibacterial activity of the plant against different pathogenic microbes (Table 5). The highest activity was observed against *Pseudomonas aeruginosa* (24.1 ± 0.3 mm) followed by *Salmonella enterica* (22.1 ± 0.1 mm). The lowest activity was observed against *E. coli* (18.5 ± 0.2 mm). The standard antibiotic gentamicin (20 µg) had growth inhibition zones of 21.7 ± 0.5, 22.1 ± 0.1, 19.7 ± 0.2, and 20.5 ± 0.2 mm against *E. coli*, *P. aeuginosa*, *S. aureus*, and *S. enterica*, respectively. Likewise, the minimum inhibitory concentration was found to be highly effective against *P. aeruginosa* (0.625 ± 0.02 mg/mL) followed by *Salmonella enterica* (1.00 ± 0.01 mg/mL). The lowest activity was observed against *E. coli* (1.50 ± 0.01 mg/mL). The MIC values of gentamicin were found to range between 0.325 and 0.625 mg/mL (Table 5).

## 3. Materials and Methods

### 3.1. Humboldtia sanjappae Collection and Extraction 

The *Humboldtia sanjappae* plant samples were collected on 12 December 2022 from Ernakulam District, Western Ghats of Kerala (10.04829° N, 76.8399° E). The mature leaves and the bark of the plants collected were carefully cleaned of all kinds of dust via washing. These materials were dried under shade for 21 days and powdered using a mixer grinder; the powder was extracted with 100% ethanol. About 5 g of each material was subjected to 6 h of extraction. All the extracts were evaporated to dryness using a rotary evaporator, and extract yield was calculated for the same.

### 3.2. Phytochemical Analysis of Humboldtia sanjappae

A preliminary phytochemical analysis of all samples was performed to determine the presence of biologically important secondary metabolites such as alkaloids, flavonoids, terpenoids, steroids, carbohydrates, saponins, etc. All tests were conducted following the conventional procedures described by Yadav, Agarwala, and Harborne [102,103]. The total phenolic content (TPC) and the total flavonoid content (TFC) were determined spectrophotometrically. TPC was found using the Folin–Ciocalteu reagent assay [104]. A standard curve was constructed using Gallic Acid standards. TPC was measured in Gallic Acid Equivalents (GAE). TFC was determined using an aluminium chloride colorimetric assay [105]. The flavonoid content was estimated using the standard quercetin calibration curve. TFC was measured in terms of quercetin equivalents. 

The LC-MS analysis was carried out according to the previous methods of House et al. [106]. Briefly, the HR-LCMS-Q-TOF analysis was carried out using Agilent 1290 UHPLC system (Agilent Technologies, Santa Clara, CA, USA). Accurately, 10 µL of the extract was injected into the system, and the run was carried out using water (0.1% formic acid *v*/*v*) (A) and methanol (B) as solvents. The gradient elution mode was used as follows: 1–10 min 95% A, 10–20 min 75% A, 20–25 min 50% A, 25–30 min 30% A, 30–40 min 95% B. The flow rate was set at 0.3 mL/min, and pressure was 1200 bar.

### 3.3. Analysis of the Antioxidant Activity of H. sanjappae Ethanol Extract

The antioxidant activities were assessed by evaluating the scavenging potentials of various radicals, such as diphenyl picryl hydrazyl (DPPH) and FRAP (Ferric Reducing Antioxidant Power). These methods allow for the measurement of the ability of the tested samples to neutralize or reduce these radicals, providing insights into their antioxidant properties [96,97]. A solution of DPPH was prepared by dissolving it in methanol at a concentration of 0.1 mM. Varying concentrations of the extract were mixed with the DPPH solution. The resulting mixture was then incubated in a dark environment at a temperature of 30 degrees Celsius for 20 min. The change in absorbance of the solution was measured and used to estimate the percentage inhibition [96]. The stock solutions consisted of a 300 mM acetate buffer (prepared by dissolving 3.1 g of sodium acetate and 16 mL of acetic acid) with a pH of 3.6, a 10 mM TPTZ (2, 4, 6-tripyridyl-s-triazine) solution (3.12 mg/mL) in 40 mM HCl, and a 20 mM FeCl3 solution (3.25 mg/mL). To prepare the fresh working solution, 25 mL of acetate buffer, 2.5 mL of TPTZ solution, and 2.5 mL of FeCl3 solution were mixed together. The resulting solution was warmed at 37 °C before use. For the FRAP assay, a test sample of varying concentrations was mixed with 2.80 mL of the prepared FRAP solution. The mixture was allowed to react for 30 min under dark conditions. After the incubation period, the absorbance of the colored product, known as the ferrous tripyridyltriazine complex, was measured at a wavelength of 593 nm [98]. The control in the experiment refers to the reaction mixture in which the test sample was not added.

### 3.4. Analysis of the H. sanjappae Ethanol Extract on Activities of Enzymes

To assess the enzyme-inhibitory properties of the test samples, specific enzymes related to diabetes and secondary diabetic complications were targeted. The inhibitory effects on α-amylase [45] and α-glucosidase [46] were examined using standard procedures.

### 3.5. Effect of H. sanjappae Ethanol Extract on Lipopolysaccharide-Induced Anti-Inflammatory Activity in Macrophages

The murine Raw 264.7 cells were seeded at 1 × 10^7^ cells/mL in a 24-well plate containing complete growth media. The *H. sanjappae* extract was diluted in RPMI-1640 media at different concentrations (5, 10, and 20 µg/mL). A standard anti-inflammatory compound, aspirin, was also used as positive control at a concentration of 1 mM. The cells were then treated with lipopolysaccharide (LPS) at a concentration of 1 μg/mL for 24 h. The levels of inflammatory cytokines such as interleukin-1β, interleukin-6, and tumor necrosis factor-α were measured using PeproTech ELISA kits. The production of nitric oxide in the media by the macrophages was quantified biochemically using the Griess method [106]. The gene expression of NF-KB and Cyclooxygenase 2 was determined by real-time PCR according to the ΔΔCT method. Briefly, the total RNA was isolated, and cDNA was synthesized using standard kits from Takara (Banglore, India). The real-time PCR analysis was carried out using the temperature cycle of 95 °C (melting temperature) for 15 s, 60 °C (annealing temperature) for 45 s, and 73 °C (extension temperature) for 30 s. The cycle was repeated 40 times, and CT values were calculated using the Applied Biosystems 7300 software. The primer sequences used in the study are listed in Supplementary Table S5.

### 3.6. Antibacterial Activity of H. sanjappae Ethanol Extract

The antibacterial activity of *H. sanjappae* was estimated in terms of the disc diffusion method according to the methods of Webber et al. [107]. The extracts were placed in circular discs and kept in the bacterial culture plate at 80 mm distance to one another. The growth inhibition zone in each of the bacterial cultures was determined and expressed as zone of inhibition in mm. The MIC value was determined according to the previous methods of Morgan et al. [108]. The gentamicin was used as a standard antibacterial agent at a concentration of 20 µg.

### 3.7. Statistical Analysis

The data were represented as mean of three independent experiments with triplicate analysis. The statistical operations were carried out using GraphPad Prism 7.0.

## 4. Conclusions

Most of the species in the genus *Humboldtia* have not been evaluated for their pharmacological potential despite their relevance in ethnomedicine. The present study for the first time reports the phytochemical composition and antioxidant, antimicrobial, and anti-inflammatory activities of *H. sanjappae*, a native of the Western Ghats of India. The study concludes that the plant has strong antioxidant properties in terms of radical scavenging and reducing potentials, and it is also effective as an antibacterial agent. Further, the extract inhibited the cytokine levels in Raw 264.7 macrophages, which is indicative of its anti-inflammatory properties. Enzymes such as α-amylase and α-glucosidase are important in controlling how our bodies absorb carbs and are frequently targeted by diabetic drugs [79]. Indeed, the potent antidiabetic actions of HSE may be connected to its capacity to inhibit α-amylase and α-glucosidase. The extract may help regulate blood sugar levels and successfully manage diabetes by inhibiting certain carbohydrate-processing enzymes. 

By carefully studying and testing, we have clearly shown that bark extract of *H. sanjappae* made with ethanol is really good at reducing inflammation, controlling diabetes, fighting bacteria, and acting as an antioxidant. These different benefits not only highlight how valuable *H. sanjappae* is, but also remind us that using plants for medicine has always been a great way to create a variety of medicines. There are many examples from history where medicinal plants have led to big changes in medicine. For example, aspirin, which comes from the bark of the willow tree (*Salix alba* L.), changed how we manage pain and helped develop other drugs such as NSAIDs (non-steroidal anti-inflammatory drugs) [109]. Additionally, the Madagascar periwinkle plant (*Catharanthus roseus* L.) gave us vinblastine and vincristine, powerful compounds that have really changed how we treat cancer [110].

Significantly, more than half of the drugs utilized worldwide in modern pharmaceuticals have their origins in natural sources [111,112]. The worldwide commercial success of established and effective pharmaceuticals taken from many plant kinds demonstrates the importance of medicinal plants as potential drug reservoirs. Quinine, an anti-malarial alkaloid derived from the bark of *Cinchona officinalis* L., is one example. Furthermore, chloroquine, derived from quinine, not only modulates inflammatory autoimmune responses but has recently shown promise in anticancer therapy [112,113].

The polypharmacological potential of *H. sanjappae*, as evidenced by its diverse array of beneficial properties, aligns perfectly with this lineage of discovery. Its capacity to simultaneously tackle a range of health factors—spanning from inflammation and diabetes to bacterial infections and oxidative stress—resonates with the holistic approach of medicinal plants. These qualities offer the potential for more complete and refined therapeutic treatments, acknowledging the complexities of human health.

## Figures and Tables

**Table 1:** LC−MS analysis and chemical composition of *H. sanjappae*.

Sl. No.	RT	*m*/*z* ^a^	*m*/*z* ^b^	Name of the Compound	Fragments	Mol. Wt.	Chemical Formula	Structure
1	3.575	577.14	577.14	Richotomine	483.13, 315.11, 197.80	532.14	C_30_H_20_N_4_O_6_	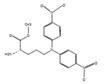
2	3.773	579.15	579.15	Procyanidin B7	579.14, 443.15, 383.12, 227.17	578.14	C_30_H_26_O_12_	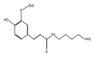
3	4.166	289.07	289.07	(−)−Epicatechin	289.07, 226.07	290.08	C_15_H_14_O_6_	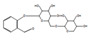
4	4.633	513.14	513.14	2″,6″−Di-O-acetylononin	513.14, 289.07	514.15	C_26_H_26_O_11_	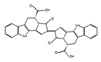
5	5.908	494.24	494.24	Ryanodine	494.23, 189.07	493.23	C_25_H_35_NO_9_	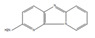
6	5.919	465.16	465.16	Pomiferin	421.16, 213.07	420.16	C_25_H_24_O_6_	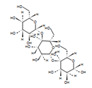
7	6.312	549.22	549.22	Cymorcin diglucoside	431.10, 253.03	490.21	C_22_H_34_O_12_	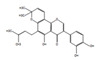
8	8.763	287.05	287.06	Maritimetin	287.05, 283.15, 267.15	286.05	C_15_H_10_O_6_	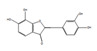
9	9.101	285.04	285.04	Luteolin	285.04, 215.09	286.05	C_15_H_10_O_6_	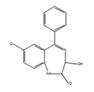
10	9.475	271.06	271.06	Naringenin	271.06, 259.12, 248.97	272.07	C_15_H_12_O_5_	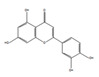
11	9.681	271.06	271.06	Coriandrone E	251.16, 179.10	248.07	C_13_H_12_O_5_	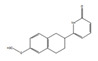
12	10.067	271.06	271.06	Morindone	271.05, 267.15, 253.14	270.05	C_15_H_10_O_5_	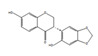
13	10.197	301.07	301.07	(+)-Sophorol	271.05, 301.06, 295.18, 277.17	300.06	C_16_H_12_O_6_	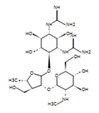
14	10.485	269.08	269.08	Formononetin	258.04, 179.10, 139.15	268.07	C_16_H_12_O_4_	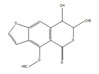
15	11.846	283.19	283.19	Lactapiperanol C	279.09, 265.17	282.18	C_16_H_26_O_4_	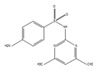
16	15.229	507.23	507.22	Limonoate	507.22, 351.25, 238.12	506.22	C_26_H_34_O_10_	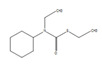
17	16.573	295.23	295.23	17−Hydroxylinolenic acid	295.22, 284.32, 277.21	294.22	C_18_H_30_O_3_	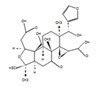
18	19.676	645.42	645.42	Capsanthin 5,6−epoxide	529.30, 403.26, 238.89	600.42	C_40_H_56_O_4_	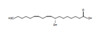
19	21.644	593.27	593.27	Ganoderic acid F	415.35, 227.17, 570.41	570.28	C_32_H_42_O_9_	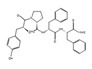
20	22.179	471.35	471.35	delta−Maslinic acid	471.85, 311.17, 248.97	472.36	C_30_H_48_O_4_	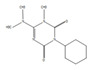
21	23.291	413.26	413.27	D8’−Merulinic acid A	391.28, 279.15, 149.02	390.28	C_24_H_38_O_4_	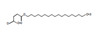

^a^: calculated m/Z ratio, ^b^: Reference m/Z ratio.

**Table 2:** The total polyphenol and flavonoid contents of *H. sanjappae* ethanol extract.

Assay	µg Equivalent/mg of Extract
Total phenolic content	378.77 ± 6.62
Total flavonoid content	204.76 ± 6.10

**Table 3:** In vitro antioxidant and antidiabetic activities of *H. sanjappae* expressed as IC_50_ values (µg/mL).

Activity	IC_50_ Value(µg/mL)		
	HSE	Ascorbic Acid	Acarbose
DPPH scavenging	6.53 ± 1.49	2.11 ± 0.25	>200
FRAP value	8.46 ± 1.38	4.15 ± 0.47	>200
α-amylase	47.60 ± 0.19	33.92 ± 2.54	122.18 ± 3.08
α-glucosidase	32.09 ± 0.54	29.85 ± 2.01	103.45 ± 2.68

**Table 4:** Anti-inflammatory activity of *H. sanjappae* extract against lipopolysaccharide-induced activation of Raw 264.7 cells and comparison with standard aspirin (1 mM).

	IL-1β (pg/mg Protein)	IL-6 (pg/mg Protein)	TNF-α (pg/mg Protein)	NO (µM/mg Protein)
Untreated	64.6 ± 1.9	133.4 ± 5.8	115.2 ± 3.1	10.7 ± 0.64
LPS Control	573.4 ± 4.5	628.5 ± 8.2	856.0 ± 11.2	75.2 ± 2.1
Aspirin (1 mM)	147.5 ± 5.1 ***	209.5 ± 9.1 ***	247.5 ± 6.3 ***	22.7 ± 1.7 ***
HSE 5 µg/mL	403.7 ± 6.2 **	507.1 ± 8.1 *	715.2 ± 8.8 *	58.8 ± 3.4 *
HSE 10 µg/mL	298.5 ± 8.4 ***	388.4 ± 2.8 ***	602.8 ± 5.2 ***	42.3 ± 1.9 ***
HSE 20 µg/mL	156.2 ± 3.4 ***	291.3 ± 6.6 ***	493.7 ± 6.4 ***	30.7 ± 2.5 ***

* indicates significant variation with respect to LPS control (*p* < 0.05); ** indicates higher significant variation with respect to that of LPS control (*p* < 0.01), and *** indicates highest significant variation with respect to that of LPS control (*p* < 0.001). All the results are indicated as mean ± standard deviation of six independent experiments.

**Table 5:** Efficacy of *H. sanjappae* (HSE) as an antimicrobial agent estimated using disc diffusion method and minimum inhibitory concentrations and comparison with gentamicin (GM).

Bacteria	Zone of Inhibition (mm)		MIC Concentration (mg/mL)	
	HSE	GM (20 µg)	HSE	GM
*Escherichia coli*	18.5 ± 0.2	21.7 ± 0.5	1.50 ± 0.01	0.325
*Pseudomonas aeruginosa*	24.1 ± 0.3	22.1 ± 0.1	0.625 ± 0.02	0.325
*Staphylococcus aureus*	20.6 ± 0.3	19.7 ± 0.2	1.25 ± 0.04	0.625
*Salmonella enterica*	22.1 ± 0.1	20.5 ± 0.2	1.00 ± 0.01	0.625

All the results are indicated as mean ± standard deviation of six independent experiments.

## Data Availability

The data may be shared upon valid request.

## Supplementary Materials

The following supporting information can be downloaded at: https://www.mdpi.com/article/10.3390/molecules28196875/s1. Table S1: Qualitative analysis of phytochemicals present in different extracts of *Humboldtia sanjappae;* Table S2: Percentage yield of different extracts of *H. sanjappae*; Table S3: Antioxidant activity of different extracts of *H. sanjappae;* Table S4: Total phenol and total flavonoid contents of different extracts of *H. sanjappae;* Table S5: The forward and reverse primer sequences of different genes used for real-time PCR analysis.

## Sample Availability

Samples of the compounds are available from the authors.

## References

[1] Kumar V., Bishayee K., Park S., Lee U., Kim J. (2023). Oxidative stress in cerebrovascular disease and associated diseases. Front. Endocrinol..

[2] Jia D., Nagaoka Y., Katsumata M., Orsulic S. (2018). Inflammation is a key contributor to ovarian cancer cell seeding. Sci. Rep..

[3] Hausmann S., Kong B., Michalski C., Erkan M., Friess H. (2014). The role of inflammation in pancreatic cancer. Inflamm. Cancer.

[4] Mastinu A., Bonini S.A., Premoli M., Maccarinelli G., Mac Sweeney E., Zhang L., Lucini L., Memo M. (2021). Protective Effects of *Gynostemma pentaphyllum* (var. Ginpent) against Lipopolysaccharide-Induced Inflammation and Motor Alteration in Mice. Molecules.

[5] Rahman S., Atikullah M., Islam M.N., Mohaimenul M., Ahammad F., Islam M.S., Saha B., Rahman H. (2019). Anti-inflammatory, antinociceptive and antidiarrhoeal activities of methanol and ethyl acetate extract of *Hemigraphis alternata* leaves in mice. Clin. Phytoscience.

[6] Golia E., Limongelli G., Natale F., Fimiani F., Maddaloni V., Pariggiano I., Bianchi R., Crisci M., D’Acierno L., Giordano R. (2014). Inflammation and cardiovascular disease: From pathogenesis to therapeutic target. Curr. Atheroscler. Rep..

[7] Iqbal J., Abbasi B.A., Mahmood T., Kanwal S., Ali B., Shah S.A., Khalil A.T. (2017). Plant-derived anticancer agents: A green anticancer approach. Asian Pac. J. Trop. Biomed..

[8] Kasote D.M., Katyare S.S., Hegde M.V., Bae H. (2015). Significance of antioxidant potential of plants and its relevance to therapeutic applications. Int. J. Biol. Sci..

[9] Oguntibeju O.O. (2018). Medicinal plants with anti-inflammatory activities from selected countries and regions of Africa. J. Inflamm. Res..

[10] Coulibaly A.Y., Hashim R., Sulaiman S.F., Sulaiman O., Ang L.Z.P., Ooi K.L. (2014). Bioprospecting medicinal plants for antioxidant components. Asian Pac. J. Trop. Med..

[11] Okach D., Nyunja A., Opande G. (2013). Phytochemical screening of some wild plants from Lamiaceae and their role in traditional medicine in Uriri District-Kenya. Int. J. Herb. Med..

[12] Liu C.H., Abrams N.D., Carrick D.M., Chander P., Dwyer J., Hamlet M.R., Macchiarini F., PrabhuDas M., Shen G.L., Tandon P. (2017). Biomarkers of chronic inflammation in disease development and prevention: Challenges and opportunities. Nat. Immunol..

[13] Nunes C.d.R., Barreto Arantes M., Menezes de Faria Pereira S., Leandro da Cruz L., de Souza Passos M., Pereira de Moraes L., Vieira I.J.C., Barros de Oliveira D. (2020). Plants as sources of anti-inflammatory agents. Molecules.

[14] Zammel N., Saeed M., Bouali N., Elkahoui S., Alam J.M., Rebai T., Kausar M.A., Adnan M., Siddiqui A.J., Badraoui R. (2021). Antioxidant and anti-Inflammatory effects of *Zingiber officinale* roscoe and *Allium subhirsutum*: In silico, biochemical and histological Study. Foods.

[15] Apaza Ticona L., Pérez-Uz B., García Esteban M.T., Aguilar Rico F., Slowing K. (2022). Anti-melanogenic and Anti-inflammatory Activities of *Hibiscus sabdariffa*. Rev. Bras. De Farmacogn..

[16] Ďuračková Z. (2010). Some current insights into oxidative stress. Physiol. Res..

[17] Rodriguez-Garcia I., Silva-Espinoza B.A., Ortega-Ramirez L.A., Leyva J.M., Siddiqui M.W., Cruz-Valenzuela M.R., Gonzalez-Aguilar G.A., Ayala-Zavala J.F. (2016). Oregano Essential Oil as an Antimicrobial and Antioxidant Additive in Food Products. Crit. Rev. Food Sci. Nutr..

[18] Mhatre S., Srivastava T., Naik S., Patravale V. (2021). Antiviral activity of green tea and black tea polyphenols in prophylaxis and treatment of COVID-19: A review. Phytomedicine.

[19] Alagumanivasagam G., Veeramani P. (2015). A review on medicinal plants with hypolipidemic activity. Int. J. Pharm. Anal. Res..

[20] Fu W., Zhuang W., Zhou S., Wang X. (2015). Plant-derived neuroprotective agents in Parkinson’s disease. Am. J. Transl. Res..

[21] Shamsudin N.F., Ahmed Q.U., Mahmood S., Shah S.A.A., Sarian M.N., Khattak M.M.A.K., Khatib A., Sabere A.S.M., Yusoff Y.M., Latip J. (2022). Flavonoids as Antidiabetic and Anti-Inflammatory Agents: A Review on Structural Activity Relationship-Based Studies and Meta-Analysis. Int. J. Mol. Sci..

[22] Gangaram S., Naidoo Y., Dewir Y.H., El-Hendawy S. (2021). Phytochemicals and Biological Activities of *Barleria* (Acanthaceae). Plants.

[23] Olivia N.U., Goodness U.C., Obinna O.M. (2021). Phytochemical profiling and GC-MS analysis of aqueous methanol fraction of *Hibiscus asper* leaves. Future J. Pharm. Sci..

[24] Njamen D., Djiogue S., Zingue S., Mvondo M.A., Nkeh-Chungag B.N. (2013). In vivo and in vitro estrogenic activity of extracts from *Erythrina poeppigiana* (Fabaceae). J. Complement. Integr. Med..

[25] Nair R.V., Jayasree D.V., Biju P.G., Baby S. (2018). Anti-inflammatory and anticancer activities of erythrodiol-3-acetate and 2, 4-di-tert-butylphenol isolated from *Humboldtia unijuga*. Nat. Prod. Res..

[26] Kumar J.K., Prasad A.D., Chaturvedi V. (2014). Phytochemical screening of five medicinal legumes and their evaluation for in vitro anti-tubercular activity. Ayu.

[27] Pavithra G., Naik A.S., Siddiqua S., Vinayaka K., TR P.K., Mukunda S. (2013). Antioxidant and antibacterial activity of flowers of *Calycopteris floribunda* (Roxb.) Poiret, *Humboldtia brunonis* Wall and *Kydia calycina* Roxb. Int. J. Drug Dev. Res..

[28] Sindhu S., Manorama S., Sumathi P., Adira S. (2014). Antimicrobial studies on the endemic medicinal plant *Humboldtia brunonis* wall. (Caesalpiniaceae). Int. J. Pharmaceut. Sci. Health Care.

[29] Asirvatham R., Yesudanam S. (2018). Neuropharmacological study of *Humboldtia vahliana* Wight. Sch. Acad. J. Pharm..

[30] Sanjappa M. (1986). A revision of the genus Humboldtia Vahl (Leguminosae-Caesalpinioideae). Blumea Biodivers. Evol. Biogeogr. Plants.

[31] Asirvatham R., Yesudanam S. (2017). Evaluation of antioxidant potential of Humboldtia Vahliana Wight in Neuropharmacological screening on mice. J. Int. Res. Med. Pharm. Sci..

[32] John B., Sulaiman C., George S., Reddy V. (2014). Total phenolics and flavonoids in selected medicinal plants from Kerala. Int. J. Pharm. Pharm. Sci..

[33] Sheik S., Chandrashekar K. (2014). Antimicrobial and antioxidant activities of *Kingiodendron pinnatum* (DC.) Harms and *Humboldtia brunonis* Wallich: Endemic plants of the Western Ghats of India. J. Natl. Sci. Found. Sri Lanka.

[34] Nisbet L.J., Moore M. (1997). Will natural products remain an important source of drug research for the future?. Curr. Opin. Biotechnol..

[35] Nagabhushan R.K., Raveesha A. (2015). Ethnobotanical survey and scientific validation of medicinal plants used in the treatment of fungal infections in Agumbe region of Western Ghats, India. Int. J. Pharm. Pharm. Sci..

[36] Vijayan A., Liju V.B., John R.J.V., Parthipan B., Renuka C. (2007). Traditional Remedies of Kani Tribes of Kottoor Reserve Forest, Agasthyavanam, Thiruvananthapuram, Kerala.

[37] Al-Khayri J.M., Sahana G.R., Nagella P., Joseph B.V., Alessa F.M., Al-Mssallem M.Q. (2022). Flavonoids as potential anti-inflammatory molecules: A review. Molecules.

[38] Ghannadi A., Hajhashemi V., Jafarabadi H. (2005). An investigation of the analgesic and anti-inflammatory effects of *Nigella sativa* seed polyphenols. J. Med. Food.

[39] Li X.-W., Chen H.-P., He Y.-Y., Chen W.-L., Chen J.-W., Gao L., Hu H.-Y., Wang J. (2018). Effects of Rich-Polyphenols Extract of Dendrobium loddigesii on Anti-Diabetic, Anti-Inflammatory, Anti-Oxidant, and Gut Microbiota Modulation in db/db Mice. Molecules.

[40] Ginwala R., Bhavsar R., Chigbu D.G.I., Jain P., Khan Z.K. (2019). Potential Role of Flavonoids in Treating Chronic Inflammatory Diseases with a Special Focus on the Anti-Inflammatory Activity of Apigenin. Antioxidants.

[41] Bettaieb A., Cremonini E., Kang H., Kang J., Haj F.G., Oteiza P.I. (2016). Anti-inflammatory actions of (−)-epicatechin in the adipose tissue of obese mice. Int. J. Biochem. Cell Biol..

[42] Reagan L.P., Magarinos A.M., McEWEN B.S. (1999). Neurological changes induced by stress in streptozotocin diabetic rats. Ann. N. Y. Acad. Sci..

[43] Freitas L.M., Antunes F.T.T., Obach E.S., Correa A.P., Wiiland E., de Mello Feliciano L., Reinicke A., Amado G.J.V., Grivicich I., Fialho M.F.P. (2021). Anti-inflammatory effects of a topical emulsion containing *Helianthus annuus* oil, glycerin, and vitamin B3 in mice. J. Pharm. Investig..

[44] Ou Z., Zhao J., Zhu L., Huang L., Ma Y., Ma C., Luo C., Zhu Z., Yuan Z., Wu J. (2019). Anti-inflammatory effect and potential mechanism of betulinic acid on λ-carrageenan-induced paw edema in mice. Biomed. Pharmacother..

[45] Kwon Y.-I.I., Vattem D.A., Shetty K. (2006). Evaluation of clonal herbs of *Lamiaceae* species for management of diabetes and hypertension. Asia Pac. J. Clin. Nutr..

[46] Shai L., Magano S., Lebelo S., Mogale A. (2011). Inhibitory effects of five medicinal plants on rat alpha-glucosidase: Comparison with their effects on yeast alpha-glucosidase. J. Med. Plants Res..

[47] Matu E.N., Van Staden J. (2003). Antibacterial and anti-inflammatory activities of some plants used for medicinal purposes in Kenya. J. Ethnopharmacol..

[48] Frieri M., Kumar K., Boutin A. (2017). Antibiotic resistance. J. Infect. Public Health.

[49] MacGowan A., Macnaughton E. (2017). Antibiotic resistance. Medicine.

[50] Alibi S., Crespo D., Navas J. (2021). Plant-derivatives small molecules with antibacterial activity. Antibiotics.

[51] Guimarães A.C., Meireles L.M., Lemos M.F., Guimarães M.C.C., Endringer D.C., Fronza M., Scherer R. (2019). Antibacterial activity of terpenes and terpenoids present in essential oils. Molecules.

[52] Simirgiotis M.J., Burton D., Parra F., López J., Muñoz P., Escobar H., Parra C. (2020). Antioxidant and antibacterial capacities of *Origanum vulgare* L. essential oil from the arid Andean Region of Chile and its chemical characterization by GC-MS. Metabolites.

[53] El Moussaoui A., Jawhari F.Z., Almehdi A.M., Elmsellem H., Benbrahim K.F., Bousta D., Bari A. (2019). Antibacterial, antifungal and antioxidant activity of total polyphenols of *Withania frutescens* L.. Bioorganic Chem..

[54] Reglodi D., Renaud J., Tamas A., Tizabi Y., Socías S.B., Del-Bel E., Raisman-Vozari R. (2017). Novel tactics for neuroprotection in Parkinson’s disease: Role of antibiotics, polyphenols and neuropeptides. Prog. Neurobiol..

[55] Ramata-Stunda A., Petriņa Z., Valkovska V., Borodušķis M., Gibnere L., Gurkovska E., Nikolajeva V. (2022). Synergistic effect of polyphenol-rich complex of plant and green propolis extracts with antibiotics against respiratory infections causing bacteria. Antibiotics.

[56] Haghjoo B., Lee L.H., Habiba U., Tahir H., Olabi M., Chu T.-C. (2013). The synergistic effects of green tea polyphenols and antibiotics against potential pathogens. Adv. Biosci. Biotechnol..

[57] Usman M., Khan W.R., Yousaf N., Akram S., Murtaza G., Kudus K.A., Ditta A., Rosli Z., Rajpar M.N., Nazre M. (2022). Exploring the phytochemicals and anti-cancer potential of the members of Fabaceae family: A comprehensive review. Molecules.

[58] Zonyane S., Fawole O.A., La Grange C., Stander M.A., Opara U.L., Makunga N.P. (2020). The implication of chemotypic variation on the anti-oxidant and anti-cancer activities of *Sutherlandia frutescens* (L.) R.Br.(Fabaceae) from different geographic locations. Antioxidants.

[59] Borquaye L.S., Doetse M.S., Baah S.O., Mensah J.A. (2020). Anti-inflammatory and anti-oxidant activities of ethanolic extracts of *Tamarindus indica* L. (Fabaceae). Cogent Chem..

[60] Abdulkhaleq L.A., Assi M.A., Noor M.H.M., Abdullah R., Saad M.Z., Taufiq-Yap Y.H. (2017). Therapeutic uses of epicatechin in diabetes and cancer. Vet. World.

[61] Dong H., Yang X., He J., Cai S., Xiao K., Zhu L. (2017). Enhanced antioxidant activity, antibacterial activity and hypoglycemic effect of luteolin by complexation with manganese (II) and its inhibition kinetics on xanthine oxidase. RSC Adv..

[62] Alshehri S., Imam S.S., Altamimi M.A., Hussain A., Shakeel F., Elzayat E., Mohsin K., Ibrahim M., Alanazi F. (2020). Enhanced dissolution of luteolin by solid dispersion prepared by different methods: Physicochemical characterization and antioxidant activity. ACS Omega.

[63] Kim N.M., Kim J., Chung H.Y., Choi J.S. (2000). Isolation of luteolin 7-O-rutinoside and esculetin with potential antioxidant activity from the aerial parts of *Artemisia montana*. Arch. Pharmacal Res..

[64] Xu H., Linn B.S., Zhang Y., Ren J. (2019). A review on the antioxidative and prooxidative properties of luteolin. React. Oxyg. Species.

[65] Guo Y., Liu Y., Zhang Z., Chen M., Zhang D., Tian C., Liu M., Jiang G. (2020). The antibacterial activity and mechanism of action of luteolin against *Trueperella pyogenes*. Infect. Drug Resist..

[66] Çetinkaya M., Baran Y. (2023). Therapeutic Potential of Luteolin on Cancer. Vaccines.

[67] Potočnjak I., Šimić L., Gobin I., Vukelić I., Domitrović R. (2020). Antitumor activity of luteolin in human colon cancer SW620 cells is mediated by the ERK/FOXO3a signaling pathway. Toxicol. Vitr..

[68] Cavia-Saiz M., Busto M.D., Pilar-Izquierdo M.C., Ortega N., Perez-Mateos M., Muñiz P. (2010). Antioxidant properties, radical scavenging activity and biomolecule protection capacity of flavonoid naringenin and its glycoside naringin: A comparative study. J. Sci. Food Agric..

[69] Patel K., Singh G.K., Patel D.K. (2018). A review on pharmacological and analytical aspects of naringenin. Chin. J. Integr. Med..

[70] Ismail N.H., Mohamad H., Mohidin A., Lajis N.H. (2002). Antioxidant activity of anthraquinones from *Morinda elliptica*. Nat. Prod. Sci..

[71] Chee C.W., Zamakshshari N.H., Lee V.S., Abdullah I., Othman R., Lee Y.K., Hashim N.M., Rashid N.N. (2022). Morindone from Morinda citrifolia as a potential antiproliferative agent against colorectal cancer cell lines. PLoS ONE.

[72] Guil-Guerrero J., Martínez-Guirado C., del Mar Rebolloso-Fuentes M., Carrique-Pérez A. (2006). Nutrient composition and antioxidant activity of 10 pepper (*Capsicum annuun*) varieties. Eur. Food Res. Technol..

[73] Sun T., Xu Z., Wu C.T., Janes M., Prinyawiwatkul W., No H. (2007). Antioxidant activities of different colored sweet bell peppers (*Capsicum annuum* L.). J. Food Sci..

[74] Ahmad M.F., Wahab S., Ahmad F.A., Ashraf S.A., Abullais S.S., Saad H.H. (2022). *Ganoderma lucidum*: A potential pleiotropic approach of ganoderic acids in health reinforcement and factors influencing their production. Fungal Biol. Rev..

[75] Dubois-Deruy E., Peugnet V., Turkieh A., Pinet F. (2020). Oxidative stress in cardiovascular diseases. Antioxidants.

[76] Ndrepepa G. (2019). Myeloperoxidase–A bridge linking inflammation and oxidative stress with cardiovascular disease. Clin. Chim. Acta.

[77] Hayes J.D., Dinkova-Kostova A.T., Tew K.D. (2020). Oxidative stress in cancer. Cancer Cell.

[78] Yaribeygi H., Sathyapalan T., Atkin S.L., Sahebkar A. (2020). Molecular mechanisms linking oxidative stress and diabetes mellitus. Oxidative Med. Cell. Longev..

[79] Alqahtani A.S., Hidayathulla S., Rehman M.T., ElGamal A.A., Al-Massarani S., Razmovski-Naumovski V., Alqahtani M.S., El Dib R.A., AlAjmi M.F. (2019). Alpha-Amylase and Alpha-Glucosidase Enzyme Inhibition and Antioxidant Potential of 3-Oxolupenal and Katononic Acid Isolated from *Nuxia oppositifolia*. Biomolecules.

[80] Semaan D.G., Igoli J.O., Young L., Marrero E., Gray A.I., Rowan E.G. (2017). In vitro anti-diabetic activity of flavonoids and pheophytins from *Allophylus cominia* Sw. on PTP1B, DPPIV, alpha-glucosidase and alpha-amylase enzymes. J. Ethnopharmacol..

[81] Feunaing R.T., Tamfu A.N., Gbaweng A.J.Y., Mekontso Magnibou L., Ntchapda F., Henoumont C., Laurent S., Talla E., Dinica R.M. (2022). In vitro Evaluation of α-amylase and α-glucosidase Inhibition of 2,3-Epoxyprocyanidin C1 and Other Constituents from *Pterocarpus erinaceus* Poir. Molecules.

[82] Facchin B.M., dos Reis G.O., Vieira G.N., Mohr E.T.B., da Rosa J.S., Kretzer I.F., Demarchi I.G., Dalmarco E.M. (2022). Inflammatory biomarkers on an LPS-induced RAW 264.7 cell model: A systematic review and meta-analysis. Inflamm. Res..

[83] Hoppstädter J., Dembek A., Linnenberger R., Dahlem C., Barghash A., Fecher-Trost C., Fuhrmann G., Koch M., Kraegeloh A., Huwer H. (2019). Toll-Like Receptor 2 Release by Macrophages: An Anti-inflammatory Program Induced by Glucocorticoids and Lipopolysaccharide. Front. Immunol..

[84] Kaneko N., Kurata M., Yamamoto T., Morikawa S., Masumoto J. (2019). The role of interleukin-1 in general pathology. Inflamm. Regen..

[85] Pyrillou K., Burzynski L.C., Clarke M.C.H. (2020). Alternative Pathways of IL-1 Activation, and Its Role in Health and Disease. Front. Immunol..

[86] Bent R., Moll L., Grabbe S., Bros M. (2018). Interleukin-1 Beta—A Friend or Foe in Malignancies?. Int. J. Mol. Sci..

[87] Chen J., Wang W., Ni Q., Zhang L., Guo X. (2022). Interleukin 6-regulated macrophage polarization controls atherosclerosis-associated vascular intimal hyperplasia. Front. Immunol..

[88] Hirani D., Alvira C.M., Danopoulos S., Milla C., Donato M., Tian L., Mohr J., Dinger K., Vohlen C., Selle J. (2022). Macrophage-derived IL-6 trans-signalling as a novel target in the pathogenesis of bronchopulmonary dysplasia. Eur. Respir. J..

[89] Rose-John S., Jenkins B.J., Garbers C., Moll J.M., Scheller J. (2023). Targeting IL-6 trans-signalling: Past, present and future prospects. Nat. Rev. Immunol..

[90] Chen J., Wei Y., Yang W., Huang Q., Chen Y., Zeng K., Chen J. (2022). IL-6: The Link Between Inflammation, Immunity and Breast Cancer. Front. Oncol..

[91] Rašková M., Lacina L., Kejík Z., Venhauerová A., Skaličková M., Kolář M., Jakubek M., Rosel D., Smetana K., Brábek J. (2022). The Role of IL-6 in Cancer Cell Invasiveness and Metastasis-Overview and Therapeutic Opportunities. Cells.

[92] Al Obeed O.A., Alkhayal K.A., Al Sheikh A., Zubaidi A.M., Vaali-Mohammed M.A., Boushey R., McKerrow J.H., Abdulla M.H. (2014). Increased expression of tumor necrosis factor-α is associated with advanced colorectal cancer stages. World J. Gastroenterol..

[93] Zhao P., Zhang Z. (2018). TNF-α promotes colon cancer cell migration and invasion by upregulating TROP-2. Oncol. Lett..

[94] Król M., Kepinska M. (2021). Human Nitric Oxide Synthase—Its Functions, Polymorphisms, and Inhibitors in the Context of Inflammation, Diabetes and Cardiovascular Diseases. Int. J. Mol. Sci..

[95] Iwata M., Inoue T., Asai Y., Hori K., Fujiwara M., Matsuo S., Tsuchida W., Suzuki S. (2020). The protective role of localized nitric oxide production during inflammation may be mediated by the heme oxygenase-1/carbon monoxide pathway. Biochem. Biophys. Rep..

[96] Liu D., Guo Y., Wu P., Wang Y., Golly M.K., Ma H. (2020). The necessity of walnut proteolysis based on evaluation after in vitro simulated digestion: ACE inhibition and DPPH radical-scavenging activities. Food Chem..

[97] Lai S.-C., Ho Y.-L., Huang S.-C., Huang T.-H., Lai Z.-R., Wu C.-R., Lian K.-Y., Chang Y.-S. (2010). Antioxidant and antiproliferative activities of *Desmodium triflorum* (L.) DC. Am. J. Chin. Med..

[98] Konaté K., Souza A., Coulibaly A., Meda N., Kiendrebeogo M., Lamien-Meda A., Millogo-Rasolodimby J., Lamidi M., Nacoulma O. (2010). In vitro antioxidant, lipoxygenase and xanthine oxidase inhibitory activities of fractions from *Cienfuegosia digitata* Cav. Sida alba L. and Sida acuta Burn f.(Malvaceae). Pak. J. Biol. Sci. PJBS.

[99] Tanaka T., Narazaki M., Kishimoto T. (2014). IL-6 in inflammation, immunity, and disease. Cold Spring Harb. Perspect. Biol..

[100] Samaraweera U., Sotheeswaran S., Uvais M., Sultanbawa S. (1983). 3,5,7,3′, 5′-Pentahydroxyflavan and 3α-methoxyfriedelan from *Humboldtia laurifolia*. Phytochemistry.

[101] Dyamavvanahalli S.L., Raveesha K.A., Nagabhushan S. (2011). Bioprospecting of selected medicinal plants for antibacterial activity against some pathogenic bacteria. J. Med. Plants Res..

[102] Yadav R., Agarwala M. (2011). Phytochemical analysis of some medicinal plants. J. Phytol..

[103] Harborne A. (1998). Phytochemical Methods a Guide to Modern Techniques of Plant Analysis.

[104] Singleton V.L., Rossi J.A. (1965). Colorimetry of total phenolics with phosphomolybdic-phosphotungstic acid reagents. Am. J. Enol. Vitic..

[105] Zhishen J., Mengcheng T., Jianming W. (1999). The determination of flavonoid contents in mulberry and their scavenging effects on superoxide radicals. Food Chem..

[106] House N.C., Puthenparampil D., Malayil D., Narayanankutty A. (2020). Variation in the polyphenol composition, antioxidant, and anticancer activity among different *Amaranthus* species. S. Afr. J. Bot..

[107] Webber D.M., Wallace M.A., Burnham C.D. (2022). Stop Waiting for Tomorrow: Disk Diffusion Performed on Early Growth Is an Accurate Method for Antimicrobial Susceptibility Testing with Reduced Turnaround Time. J. Clin. Microbiol..

[108] Morgan B.L., Depenbrock S., Martínez-López B. (2022). Identifying Associations in Minimum Inhibitory Concentration Values of *Escherichia coli* Samples Obtained From Weaned Dairy Heifers in California Using Bayesian Network Analysis. Front. Vet. Sci..

[109] Di Simone S.C., Acquaviva A., Libero M.L., Chiavaroli A., Recinella L., Leone S., Brunetti L., Politi M., Giannone C., Campana C. (2022). The association of *Tanacetum parthenium* and *Salix alba* extracts reduces cortex serotonin turnover, in an ex vivo experimental model of migraine. Processes.

[110] Iskandar N.N., Iriawati I. (2016). Vinblastine and Vincristine production on Madagascar Periwinkle (*Catharanthus roseus* (L.) G. Don) callus culture treated with polethylene glycol. Makara J. Sci..

[111] Rao P., Knaus E.E. (2008). Evolution of nonsteroidal anti-inflammatory drugs (NSAIDs): Cyclooxygenase (COX) inhibition and beyond. J. Pharm. Pharm. Sci..

[112] Khumalo G.P., Van Wyk B.E., Feng Y., Cock I.E. (2022). A review of the traditional use of southern African medicinal plants for the treatment of inflammation and inflammatory pain. J. Ethnopharmacol..

[113] Bharadwaj K.C., Gupta T., Singh R.M. (2018). Alkaloid group of *Cinchona officinalis*: Structural, synthetic, and medicinal aspects. Synthesis of Medicinal Agents from Plants.

