# Supplementary material for: Phytochemical Composition and Antioxidant and Anti-Inflammatory Activities of Humboldtia sanjappae Sasidh. & Sujanapal, an Endemic Medicinal Plant to the Western Ghats
Source: Molecules. 2023 Sep 29;28(19):6875. doi: 10.3390/molecules28196875 (PMC10574196; doi:10.3390/molecules28196875)
Supplement: Supplementary file 1 [file molecules-28-06875-s001.zip › molecules-2562307-supplementary.pdf]

Table S1. Qualitative analysis of phytochemicals present in different extracts of *Humboldtia sanjappae*

Test	HBA	HBB	HBC	HBD	HBE	HLA	HLB	HLC	HLD	HLE
**Alkaloid**										
Mayer's test	–	+	–	–	+	+	+	+	–	–
Wagner's test	–	+	+	+	–	++	+	+	+	–
Dragendroff's test	–	–	+	–	–	++	++	+	+	–
Hager's test	–	–	–	–	–	++	+	+	+	–
**Carbohydrate**										
Molisch's test	++	++	++	++	+	+++	++	++	++	+
Fehling's tets	+++	++	++	+	+	+++	++	++	++	+
Bendict's test	+++	++	++	++	+	++	++	+	+	+
**Proteins**										
Biuret test	+	+	+	+	+	+	+	+	+	+
ninhydrin test	+	+	+	+	+	+	+	+	+	+
**Phenolics and Tannins**										
Ferric Chloride	+	+	+	+	+	+	+	+	+	+
Lead Acetate	+	+	+	+	+	+	+	+	+	+
**Flavones**										
Sodium hydrochloride test	++	++	++	++	++	++	++	+	+	+
Sulphuric acid test	+	+	+	+	+	+	+	+	+	+
Schinoda test	+	+	+	+	+	+	+	+	+	+
**Sterols**										
Libermann's	+	+	–	–	–	+	+	+	–	–
Liber buchards	+	–	–	–	–	+	+	+	–	–
**Triterpenoid**	+++	+	+	–	–	+++	+	+	–	–
**Saponins**										
Foam test	+++	++	++	+	+	+++	++	++	++	+

+++ strongly present, ++ present, +weekly present, – absent

HBA-*Humboldtia sanjappae* Bark extract made using 100% alcohol, HBB-*H. sanjappae* Bark extract using 80 % alcohol and 20% water, HBC-*H. sanjappae* Bark extract using 70 % alcohol and 30% water, HBD-*H. sanjappae* Bark extract using 50 % alcohol and 50% water, HBE-*H. sanjappae* Bark extract using 100% water, HLA-*H. sanjappae* leaf extract made using 100% alcohol, HBB-*H. sanjappae* Leaf extract using 80 % alcohol and 20% water, HBC-*H. sanjappae* leaf extract using 70 % alcohol and 30% water, HBD-*H. sanjappae* leaf extract using 50 % alcohol and 50% water, HBE-F leaf extract using 100% water

Table S2. Percentage Yield of Different Extracts of *H. sanjappae*

Samples	Extract Yield (%)
**HBA**	**16**
**HBB**	**14**
**HBC**	**14.2**
**HBD**	**18.2**
**HBE**	**16.2**
**HLA**	**12**
**HLB**	**13.4**
**HLC**	**15.4**
**HLD**	**12.6**
**HLE**	**17.8**

HBA-*Humboldtia sanjappae* Bark extract made using 100% alcohol, HBB-*H. sanjappae* Bark extract using 80 % alcohol and 20% water, HBC-*H. sanjappae* Bark extract using 70 % alcohol and 30% water, HBD-*H. sanjappae* Bark extract using 50 % alcohol and 50% water, HBE-*H. sanjappae* Bark extract using 100% water, HLA-*H. sanjappae* leaf extract made using 100% alcohol, HBB-*H. sanjappae* Leaf extract using 80 % alcohol and 20% water, HBC-*H. sanjappae* leaf extract using 70 % alcohol and 30% water, HBD-*H. sanjappae* leaf extract using 50 % alcohol and 50% water, HBE-F leaf extract using 100% water

Table S3. Antioxidant activity of different extract of *H. sanjappae*

Samples	DPPH Activity **IC_{50}(μg/mL)**	FRAP Activity EC50(**μg/mL)**
HBA	6.52 ± 1.49	4.33±1.65
HBB	35.48 ± 1.93	4.40±.26
HBC	30.30± 3.05	4.11±0.05
HBD	14.44± 1.68	5.93±.30
HBE	184.42± 19.40	19.41±1.02
HLA	40.87± 1.295	16.40±.80
HLB	46.03± 2.59	13.66±.58
HLC	58.87± 2.07	11.57±.56
HLD	76.35± 6.93	10.22±.42
HLE	252.24± 2.63	26.52±1.99

Data Represented as ± standard deviation, HBA-*Humboldtia sanjappae* Bark extract made using 100% alcohol, HBB-*H. sanjappae* Bark extract using 80 % alcohol and 20% water, HBC-*H. sanjappae* Bark extract using 70 % alcohol and 30% water, HBD-*H. sanjappae* Bark extract using 50 % alcohol and 50% water, HBE-*H. sanjappae* Bark extract using 100% water, HLA-*H. sanjappae* leaf extract made using 100% alcohol, HBB-*H. sanjappae* Leaf extract using 80 % alcohol and 20% water, HBC-*H. sanjappae* leaf extract using 70 % alcohol and 30% water, HBD-*H. sanjappae* leaf extract using 50 % alcohol and 50% water, HBE-F leaf extract using 100% water

Table S4. Total phenol and total flavonoid contents of different extracts of *H. sanjappae*

**Extract**	**Total Phenolic Contents (µg GAE/ mg extract)**	**Total Flavonoid Contents (µg QE/ mg extract)**
HBA	378.77±6.62	204.76 ± 6.10
HBB	411.51±6.41	278.14 ±4.57
HBC	399.14±4.63	361.25± 6.88
HBD	342.98±5.69	191.13± 5.27
HBE	296.82±5.95	201.57± 4.96
HLA	165.54±5.53	130.96± 8.56
HLB	216.48±6.99	157.83± 6.00
HLC	215.57±1.68	113.81±5.49
HLD	235.35±5.42	154.32± 7.61
HLE	123.17±5.28	78.11±2.74

Table S5. The forward and reverse primer sequences of different genes used for real-time PCR analysis

Gene	Forward/ Reverse	Sequence
Cyclooxygenase 2 (COX2)	F	5’-ATCATAAGCGAGGACCTGGG-3’
	R	5’-TCAGGGATGTGAGGAGGGTA-3’
NF-kappa-B p65	F	5′-CATACGCTGACCCTAGCCTG-3′
	R	5′-TTTCTTCAATCCGGTGGCGA-3′
Beta actin	F	5’-TCACCCACACTGTGCCCATCTACGA-3’
	R	5’-GGATGCCACAGGATTCCATACCCA-3’
